# Chronotype Genetic Variant in *PER2* is Associated with Intrinsic Circadian Period in Humans

**DOI:** 10.1038/s41598-019-41712-1

**Published:** 2019-03-29

**Authors:** Anne-Marie Chang, Jeanne F. Duffy, Orfeu M. Buxton, Jacqueline M. Lane, Daniel Aeschbach, Clare Anderson, Andrew C. Bjonnes, Sean W. Cain, Daniel A. Cohen, Timothy M. Frayling, Joshua J. Gooley, Samuel E. Jones, Elizabeth B. Klerman, Steven W. Lockley, Mirjam Munch, Shantha M. W. Rajaratnam, Melanie Rueger, Martin K. Rutter, Nayantara Santhi, Karine Scheuermaier, Eliza Van Reen, Michael N. Weedon, Charles A. Czeisler, Frank A. J. L. Scheer, Richa Saxena

**Affiliations:** 1 Department of Biobehavioral Health, Pennsylvania State University, University Park, Pennsylvania 16802 USA; 2 Division of Sleep and Circadian Disorders, Department of Medicine and Department of Neurology, Brigham and Women’s Hospital, Boston, Massachusetts 02115 USA; 3 Division of Sleep Medicine, Harvard Medical School, Boston, Massachusetts 02115 USA; 4 Medical and Population Genetics, Broad Institute of Harvard and Massachusetts Institute of Technology, Cambridge, Massachusetts 02142 USA; 5 Department of Social and Behavioral Sciences, Harvard Chan School of Public Health, Boston, Massachusetts 02115 USA; 6 Department of Anesthesia, Critical Care and Pain Medicine and Center for Genomic Medicine; Massachusetts General Hospital, Boston, Massachusetts 02114 USA; 7 Department of Sleep and Human Factors Research, Institute of Aerospace Medicine, German Aerospace Center, Cologne, 51147 Germany; 8 Genetics of Complex Traits, University of Exeter Medical School, Exeter, United Kingdom; 9 Division of Endocrinology, Diabetes & Gastroenterology, School of Medical Sciences, Faculty of Biology, Medicine and Health, University of Manchester, Manchester, UK; 10 Manchester Diabetes Centre, Manchester University NHS Foundation Trust, Manchester Academic Health Science Centre, Manchester, UK; 11 Present Address: Monash Institute of Cognitive and Clinical Neurosciences and School of Psychological Sciences, Monash University, Clayton, VIC Australia; 12 Present Address: Program in Neuroscience and Behavioral Disorders, Duke-NUS Medical School, Singapore, Singapore; 13 Present Address: Sleep/Wake Research Centre, College of Health, Massey University, Wellington, New Zealand; 14 Present Address: Surrey Sleep Research Centre, University of Surrey, Guildford, UK; 15 Present Address: Wits Sleep Laboratory, Brain Function Research Group, School of Physiology, Faculty of Health Sciences, University of the Witwatersrand, Johannesburg, South Africa; 16 Present Address: Department of Psychiatry and Human Behavior, Alpert Medical School of Brown University, Providence, RI USA

## Abstract

The *PERIOD2* (*PER2*) gene is a core molecular component of the circadian clock and plays an important role in the generation and maintenance of daily rhythms. Rs35333999, a missense variant of *PER2* common in European populations, has been shown to associate with later chronotype. Chronotype relates to the timing of biological and behavioral activities, including when we sleep, eat, and exercise, and later chronotype is associated with longer intrinsic circadian period (cycle length), a fundamental property of the circadian system. Thus, we tested whether this *PER2* variant was associated with circadian period and found significant associations with longer intrinsic circadian period as measured under forced desynchrony protocols, the ‘gold standard’ for intrinsic circadian period assessment. Minor allele (T) carriers exhibited significantly longer circadian periods when determinations were based on either core body temperature or plasma melatonin measurements, as compared to non-carriers (by 12 and 11 min, respectively; accounting for ~7% of inter-individual variance). These findings provide a possible underlying biological mechanism for inter-individual differences in chronotype, and support the central role of *PER2* in the human circadian timing system.

## Introduction

The *Period* gene was the first circadian gene discovered by Konopka and Benzer in *Drosophila*^1^ and the central gene on which the concept of a transcriptional-translational feedback loop was based^2^. Mammalian homologues, including *PER2*, were found to be expressed in the suprachiasmatic nucleus (SCN)^3^, and have been shown to influence circadian period^4,5^. The other two mammalian homologues, *PER1* and *PER3*, have been reported to associate with measures indirectly related to circadian period, i.e., circadian phenotypes and chronotype^6–11^. Together, these studies highlight the central role of the *Period* genes and specifically *PER2* in the generation and maintenance of circadian rhythmicity and timing. However, to date, none of the PERs have been shown to be related to circadian period measured under the ‘gold standard’ forced desynchrony conditions in humans, necessary and designed to uncover the endogenous circadian period independent of the dark/light, sleep/wake, fasting/feeding, and other behavioral influences.

A *PER2* variant, rs35333999 (p.Val903Ile), which is common in people of European ancestry but rare in African and East Asian populations (https://www.ncbi.nlm.nih.gov/snp/rs35333999#frequency_tab), was recently associated with chronotype in a large genome-wide association study (GWAS)^12^ showing a more evening-type preference in carriers of the minor allele (T) than in those with the non-T allele (C/C) genotype. Chronotype is genetically influenced, but previous studies using a candidate-gene approach exhibited limited replicability^7,8,13–22^. More recently, GWAS have identified associations of single nucleotide polymorphisms with chronotype using an unbiased approach, as opposed to candidate gene testing, in large cohorts (https://www.ncbi.nlm.nih.gov/pubmed/30696823)^12,23^. These studies have identified several genetic associations in genomic regions harboring circadian genes, including genes *PER2*, *PER3*, *Vasoactive intestinal peptide* (*VIP*), and *F-Box and Leucine Rich Repeat Protein 3* (*FBXL3*), which is consistent with the hypothesis that common variation influencing circadian genes impacts on phenotypic chronotype. Defining the causal variant and causal gene will require additional work, however. Importantly, *PER2* rs35333999 represents the strongest association signal that peaks within a circadian gene^12^. This *PER2* variant encodes a missense single nucleotide polymorphism (SNP) in exon 19 in the canonical transcript, is conserved across mammalian species, and is predicted to be deleterious to the PER2 protein (probably damaging, score 0.963)^24^. Furthermore, the SNP lies in the 3′UTR of a non-canonical *PER2* transcript and may also have a regulatory role, as it is predicted to alter several transcription factor binding sites^25^. It is unknown, however, whether this missense variant is causal or simply a marker in linkage disequilibrium with a known or unknown causal variant. Notably, this variant is in strong linkage disequilibrium with regulatory variants rs77939198 (pair-wise r^2^ = 0.83, D’ = 0.94) and rs960783 (pair-wise r^2^ = 0.64, D’ = 0.94), both of which are annotated with chromatin marks and protein binding sites from the Roadmap Epigenomics and Encyclopedia of DNA Elements (ENCODE) projects^25^. We therefore hypothesized that this polymorphism would affect intrinsic circadian period, in addition to correlated phenotypes chronotype, sleep timing, circadian phase, and circadian phase angle according to genotype in this study.

## Results

We tested the association of *PER2* rs35333999 (p.Val903Ile) with self-reported chronotype (morningness-eveningness preference) in the full UK Biobank dataset of unrelated individuals of European ancestry (n up to 335,789), extending the sample size of our previous genome-wide association analysis based on an interim release of this dataset^12^ by up to 235,369 additional individuals. We observed genome-wide significant association of rs35333999 with morningness-eveningness preference; with the rare rs35333999 T allele conferring increased eveningness in analysis of categorical chronotype (p = 10^−14^) and dichotomized extremes (p = 10^−9^; Table 1, Supplemental Fig. 1). This larger analysis confirms the SNP association seen in the previous study^12^, as the statistical evidence for categorical chronotype strengthened from p = 10^−8^ in the nested smaller sample (n = 100,420) to p = 10^−14^ in the current sample (n = 335,789), with similar effect estimates. Conditional association analysis adjusting for the primary association signal in this genomic region (represented by SNP rs80271258) confirmed that the association of *PER2* rs35333999 with chronotype is independent of the primary signal and itself robust (Table 1, Supplemental Fig. 2).

**Table 1:** Results from association testing of *PER2* rs35333999 and regional lead SNP rs80271258 with chronotype in unrelated UK Biobank participants of white British ancestry.

SNP	E/A	Categorical Chronotype (n = 335,789) Beta (SE)	p	Extreme Chronotype* (n = 26,056 cases, 80,065 controls) OR (95% CI)	p
rs35333999	T/C	0.058 (0.008)	**9.7** × **10**^**−14**^	1.16 (1.10–1.22)	**1.5** × **10**^**−9**^
rs35333999 conditioned on rs80271258	T/C	0.063 (0.008)	**6.0** × **10**^**−16**^	1.18 (1.12–1.23)	**4.6** × **10**^**−11**^
rs80271258	T/C	0.055 (0.006)	**3.5** × **10**^**−23**^	1.14 (1.10–1.18)	**5.1** × **10**^**−13**^
rs80271258 conditioned on rs35333999	T/C	0.058 (0.006)	**2.7** × **10**^**−25**^	1.15 (1.11–1.19)	**2.0** × **10**^**−14**^

E/A = effect/alternative allele. Beta and standard error (SE) derive from a genetic regression model showing per allelic effect of rs35333999 and rs80271258 on chronotype. SNPs rs80271258 and rs35333999 are uncorrelated in European populations (r^2^ < 0.01). Significant results (p < 5 × 10^−8^) are shown in bold. Results from association testing of rs35333999 and rs80271258 conditioned on the other SNP demonstrate independence of both association signals.

^*^Evening types coded as cases and morning types as controls.

**Figure 1:**
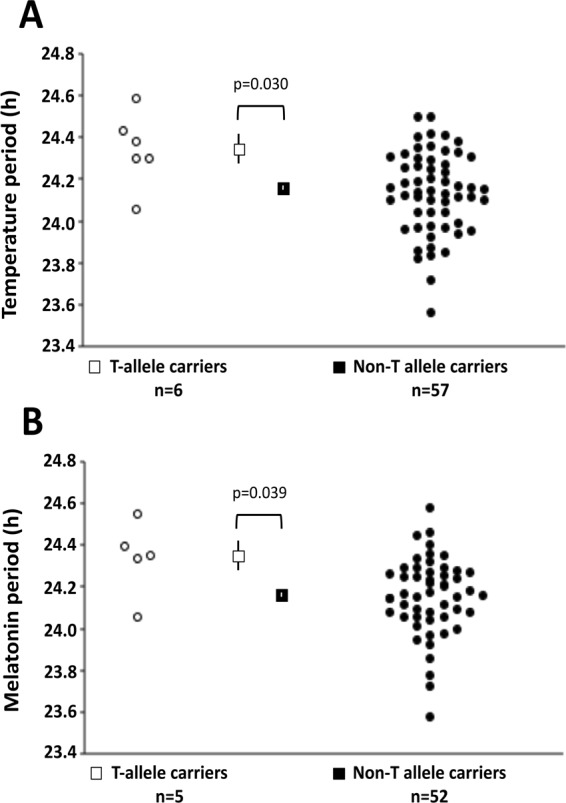
Circadian period of core body temperature and melatonin rhythms by *PER2* rs35333999 genotype in in-laboratory study participants of European ancestry. Data for mean (squares) and individual (circles) circadian period of core body temperature (**A**) and melatonin (**B**) are shown for T-allele carriers (open symbols) and for non-T allele carriers (filled symbols). Vertical lines denote the standard error (SE) measures of the mean. T-allele carriers had significantly longer temperature and melatonin circadian periods than non-carriers (p = 0.030 and 0.039, respectively).

**Figure 2:**
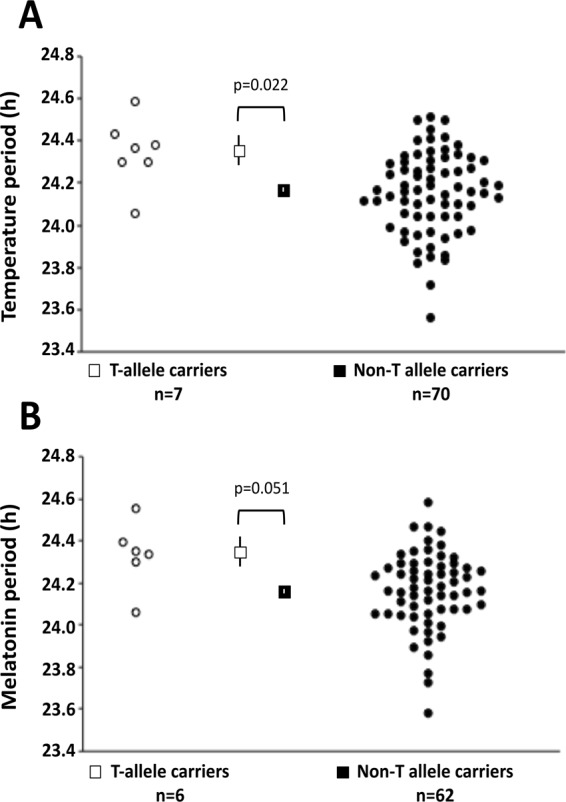
Circadian period of CBT and melatonin rhythms by *PER2* rs35333999 genotype in the multiethnic in-laboratory study sample. Data for mean (squares) and individual (circles) circadian period of core body temperature (**A**) and melatonin (**B**) are shown for T-allele carriers (open symbols) and for non-T allele carriers (filled symbols). Vertical lines denote the standard error (SE) measures of the mean. T-allele carriers had significantly longer circadian period of temperature (p = 0.022) and a trend for longer circadian period of melatonin (p = 0.051) than non-carriers.

To discern underlying mechanisms, we tested the association of *PER2* rs35333999 with circadian phenotypic measures in 196 healthy men and women of European ancestry who participated in highly controlled inpatient physiological protocols. Demographics, circadian measures and results of association testing from these participants are presented in Table 2 and Fig. 1 and results from the entire multiethnic sample (up to n = 252) are presented in Fig. 2, Supplemental Table 1 and Supplemental Fig. 3. Circadian phenotypes included chronotype (n = 193), determined by morningness-eveningness questionnaire (MEQ)^26^; intrinsic period of endogenous circadian rhythms of core body temperature (CBT; n = 63) and plasma melatonin (n = 57); circadian phase of endogenous CBT (n = 90) and melatonin (n = 102) rhythms; measures of sleep timing while on a self-selected, fixed, 8-hour sleep schedule one week prior to inpatient studies (n = 152); and phase angle between endogenous circadian phase markers and sleep timing for CBT (n = 88) and melatonin (n = 100).

**Table 2:** Phenotype measures by *PER2* rs35333999 genotype and results from association testing for in-laboratory participants of European ancestry.

Phenotype	n	Mean (SD)	n	Mean T Carriers	n	Mean Non-T Carriers	Beta (SE)	p
Age (years)	196	26.93 (11.16)	13	26.15 (5.11)	183	26.99 (11.48)	—	—
Sex (M/F)	196	126/70	13	8/5	183	118/65	—	—
MEQ score*	193	52.29 (12.14)	12	47.17 (15.27)	181	52.63 (11.88)	−3.87 (3.08)	0.210
CBT period (h)	63	24.16 (0.20)	6	24.34 (0.17)	57	24.14 (0.20)	0.20 (0.09)	**0.030**
MEL period (h)	57	24.17 (0.19)	5	24.34 (0.18)	52	24.15 (0.19)	0.19 (0.09)	**0.039**
CBT phase (clock h)	90	5:00 (2:07)	6	4:53 (1:58)	84	5:01 (2:08)	0.19 (0.91)	0.836
MEL phase (clock h)	102	22:25 (1:52)	5	22:49 (1:32)	97	22:24 (1:53)	0.67 (0.82)	0.415
CBT phase angle (h)	88	−3.19 (1.33)	6	−2.88 (1.08)	82	−3.22 (1.35)	0.55 (0.60)	0.360
MEL phase angle (h)	100	−9.49 (1.21)	5	−9.06 (1.10)	95	−9.51 (1.21)	0.57 (0.57)	0.304
Bedtime (clock h)	152	23:57 (1:27)	9	0:17 (1:44)	143	23:56 (1:26)	0.47 (0.47)	0.315
Wake time (clock h)	152	7:59 (1:27)	9	8:17 (1:41)	143	7:58 (1:26)	0.42 (0.47)	0.380
TIB duration (h)	152	8.04 (0.13)	9	7.99 (0.16)	143	8.05 (0.13)	−0.06 (0.05)	0.222
TIB midpoint (clock h)	152	3:58 (1:27)	9	4:17 (1:43)	143	3:57 (1:26)	0.44 (0.47)	0.346

Phenotype means (standard deviation; SD) and the number of males/females are listed by rs35333999 genotype: T-allele carriers and non-T carriers. Beta and standard error (SE) derive from a genetic regression model showing per allelic effect of rs35333999 on circadian phenotypes, adjusted for age, sex and PCs. Significant results (p < 0.05) are shown in bold. Circadian phenotypes include MEQ; circadian period, circadian phase and phase angle of CBT and melatonin (MEL). Sleep timing measures include bedtime, wake time, time-in-bed duration (TIB), and midpoint of TIB. Sleep phenotypes included in this analysis were assessed using call-in data during a fixed 8-hour schedule maintained the week prior to admission. ^*^A higher MEQ score reflects more morningness^26^.

### Circadian period

The *PER2* polymorphism was associated with intrinsic circadian period of the endogenous rhythms of core body temperature and circulating melatonin concentrations after adjustment for age, sex and population structure using 5 principal components of ancestry (Table 2 and Fig. 1). T-allele carriers exhibited a significantly longer intrinsic circadian period than non-T carriers for CBT (mean ± SE; 24.34 ± 0.17 h vs. 24.14 ± 0.20 h; p = 0.030) and melatonin (24.34 ± 0.18 h vs. 24.15 ± 0.19 h; p = 0.039). This represents a mean difference of 0.20 hours (12 minutes) for CBT period and a mean difference of 0.19 hours (11 minutes) for melatonin period between genotype groups and represents 7.1% of population variance in temperature period and 6.9% of variance in melatonin period in participants of European ancestry from our sample. Results were similar in the full multiethnic sample (see Fig. 2 and Supplemental Table 1). Sensitivity analysis using the unequal variances t-test to account for unequal sample size (Welch’s t-test for rs35333999 T carriers vs CC) shows a consistent direction of effect in those of European ancestry (melatonin period p = 0.189; CBT period p = 0.097) and reached significance for temperature period in the multiethnic sample (melatonin period p = 0.053; CBT period p = 0.032).

### Chronotype

Analysis of rs35333999 with composite MEQ score showed no significant association in the European-only group (p = 0.210; Table 2) or in the full multiethnic sample (p = 0.120; Supplemental Table 1 and Supplemental Fig. 3). Given the magnitude of the effect observed in the large UK Biobank GWAS study (n = 100,420)^12^, statistical power to detect the association with chronotype in the current analysis was only 6.2% in participants of European ancestry, and only 6.7% in the multi-ethnic sample.

## Discussion

The T-allele of *PER2* rs35333999 was associated with self-reported evening chronotype in a population-scale sample and with longer intrinsic circadian period as assessed in highly controlled in-laboratory circadian protocols. The consistent direction and magnitude of effect on intrinsic circadian period based on two endogenous circadian phase markers, CBT and circulating plasma melatonin rhythms, suggests a difference in the intrinsic period of the master circadian pacemaker located in the hypothalamic suprachiasmatic nucleus (SCN). The significant association between the minor allele of rs35333999 and longer period of body temperature and plasma melatonin is also consistent with published reports showing an association of evening chronotype with longer circadian period^27,28^.

This is the first report of a genetic association with intrinsic circadian period directly measured in humans using forced desynchrony protocols, specifically designed to precisely determine this physiological measure (see Methods section)^29,30^. A previous study reported genetic linkage between another *PER2* marker (D2S395) and Advanced Sleep Phase Disorder in a multi-generational family^31^. This very rare mutation (minor allele frequency <0.1%) that causes a serine to glycine change leading to hypophosphorylation of PER2 protein, was found to be associated with an early circadian timing of sleep, extreme morning chronotype, and a shorter observed period of the sleep-wake and temperature cycles in one individual, though intrinsic period free of influence of self-selected light exposure was not assessed in that study (https://www.ncbi.nlm.nih.gov/pubmed/10470086). In the current study, a relatively common variant of *PER2* (5% allele frequency in the 1000 Genomes European population and 3.6% in our population of European ancestry) was associated with intrinsic circadian period assessed under conditions free from the influence of self-selected light exposure. This was achieved by studying participants under dim light conditions (<15 lux), and by distributing that light exposure across all circadian phases outside the range of entrainment of the human circadian pacemaker. This was done in order to minimize the effects of light on circadian period. These conditions are critical for accurate assessment of intrinsic circadian period, as retinal light exposure has a powerful resetting effect on the human circadian pacemaker that would otherwise confound assessment of intrinsic circadian period^32^. Furthermore, because there are aftereffects of prior light exposure on circadian period^33^, we tried to limit differences in prior history by excluding recent shiftwork and transatlantic flights, and had participants maintain a fixed 8-h time in bed for the weeks prior to admission to the laboratory, thereby standardizing the prior light history.

The main strength of our study is the precise assessment of endogenous circadian measures in carefully designed circadian protocols conducted under rigorously controlled laboratory conditions that are considered “gold standard” procedures for determination of circadian period and phase in humans. While chronotype is a measure that is relatively easily obtainable using a questionnaire, and therefore scalable and reflects people’s preferences, it provides less insight into the relative contribution of environmental/behavioral factors (including school, work, and other social demands, exposure to light, caffeine, buildup and discharge of sleep pressure, and social demands) *versus* biology. Furthermore, chronotype cannot distinguish among different biological causes, such as differences in endogenous circadian period, circadian rhythm robustness, circadian light sensitivity, and homeostatic sleep regulation time constants. For example, the recent finding that *PER2* is associated with reduced sensitivity of the circadian pacemaker to light in humans^34^, highlights the relative benefits of biological measures of circadian rhythmicity in studying the potential mechanisms underlying chronotype differences. Circadian period provides a plausible biological mechanism of the link between the *PER2* variant and chronotype, and also provides testable hypotheses for future molecular mechanism studies in animal and *in vitro* models. Intrinsic circadian period is a key factor determining behavioral timing. A longer circadian period, leading to a delay of the circadian timing system–including the circadian drive for wake and sleep–is a plausible pathway to a later chronotype. Thus, to assess the most proximal biological mechanism, we focused specifically on intrinsic circadian period. The second reason to focus on intrinsic circadian period as a potential mechanism underlying the link between the *PER2* variant and chronotype is that in rodent mutant models, *PER2* has been shown to play a key role in determining circadian period of the rest activity cycle^4,5,31^. A third reason to focus primarily on circadian period, as opposed to the downstream measures of circadian phase (i.e., timing of circadian markers such as core body temperature and melatonin), sleep timing, circadian phase angle (i.e., relative timing of circadian markers and sleep timing), and chronotype, is that intrinsic circadian period as assessed under forced desynchrony protocols can be determined with great precision^29^, enhancing the statistical power for this outcome measure. This may be why those other circadian measures did not show significant differences, though they trended consistently later in the T-allele carriers than in the non-carriers, consistent with a longer period^27,35^.

While significant associations between rs35333999 and circadian period were identified because of a large effect (Cohen’s d was 1.08 for CBT period and 1.03 for melatonin period), we likely did not see significant associations of the SNP with circadian phase due to the overall smaller effect (Cohen’s d was 0.07 for CBT phase and 0.22 for melatonin phase). One possible explanation is that circadian period is a more proximal phenotype, i.e., one that is influenced directly by the *PER2* SNP, while phase angle is a measure that is influenced by circadian period, but also by exposure to photic and non-photic *Zeitgebers* (time cues), as well as the accumulation and dissipation of homeostatic sleep pressure^36^. Another potential explanation involves the assessment of sleep timing during the week prior to the inpatient protocols in participants who maintained a self-selected, 8-hour sleep duration schedule (see Methods section). Therefore, we were unable to assess the influence of rs35333999 on unrestricted sleep timing measures or other circadian phenotypes that incorporate them (i.e., phase angle), and may explain why there was no significant association with these phenotypes. Further work assessing this SNP in larger datasets would be necessary to test this. We also did not replicate the association of *PER2* rs35333999 with chronotype in the current study. The lack of a significant effect on chronotype was likely due to insufficient power in this sample (n = 246) relative to the UK Biobank nested sample (n = 100,420)^12^ or the larger sample (n = 335,789) in the current analysis. Another explanation could be that, similar to phase, chronotype is a less proximal phenotype than circadian period and influenced by other factors. While here we show an association of the *PER2* SNP rs35333999 with human endogenous circadian period, future studies are needed to test whether this SNP is the causal variant. Such verification will require further experimental studies (e.g., *ex vivo* cell culture recordings^37^, or phenotype rescue in animal models)^38^. Furthermore, further studies are needed to test whether this SNP may also relate to homeostatic sleep regulation, which has also associates with chronotype^36^.

Although rs35333999 is associated with self-reported chronotype^12^ and intrinsic circadian period as shown here, it is not known if this is the causal variant, or what effect, if any, the allele may have on cell-autonomous circadian rhythms. This missense SNP is predicted to have deleterious effects on PER2 protein and is also predicted to play a role in the circadian molecular mechanism by potentially altering transcription factor binding sites^24^. Future studies are needed to determine the molecular and cellular mechanism by which rs35333999 affects circadian period. Different research approaches for testing the effect of genetic variants have different strengths and limitations. The use of self-reported data collected in humans under ‘free living conditions’, as is the case for the association of the SNP with self-reported chronotype in the UK Biobank, have the benefit that these assessments can be conducted in large scale studies and have the largest generalizability to the human condition. Such populations can be useful for discovery of genetic variants influencing behavioral preferences. The downside is that they cannot assess physiological mechanisms. The benefit of the association of a SNP with an accurately and precisely assessed physiological phenotype, as is the case for endogenous circadian period under the forced desynchrony protocol, is that the physiological mechanism can be tested. The molecular mechanism, however, cannot be determined in these studies and requires genetic manipulation studies *in vivo*, or *in vitro*. The systemic integration of the effect of the SNP on the physiology can only be assessed in an *in vivo* system, while the physiological circadian mechanism in humans can only accurately be tested in human circadian protocols. The combination of these approaches will give a complete picture of the molecular and physiological mechanisms as well as the relevance in humans. Thus, ultimately, *in vitro* experiments are needed to test the influence of rs35333999 on molecular mechanisms, including potential alteration of the role of the PER2 protein in the repressor complex of the circadian clock and its translocation to the nucleus^39^.

### Limitations

Genetic analyses of the in-laboratory population included a small sample size of rs35333999 minor-allele (T) carriers for several circadian measures. This was due to the relatively low frequency of this variant, and the intensive nature of the studies required to obtain accurate and precise assessments of human intrinsic circadian period. Despite the limited sample size, we found significant differences of large magnitude in intrinsic circadian period, both when using core body temperature and plasma melatonin assessments during forced desynchrony protocols. Future studies in larger populations, including other genetic ancestries, are needed to verify and expand this finding. As previously noted, we did not find an association between rs35333999 and chronotype, as assessed by composite MEQ score, in the in-laboratory sample. The MEQ has been widely used as a validated measure of diurnal preference and it has been shown that the MEQ, the reduced MEQ (rMEQ) and the Composite Scale of Morningness (CSM) have high correlative validity^40^; however, these and other scales do not necessarily measure the same thing, especially in a cross-cultural context. For example, in a sample of Korean adults, the global CSM score as well as two of three subscales (“morningness” and “activity planning”) were associated with a non-synonymous *PER2* SNP rs934945^20^. Interestingly, this same *PER2* SNP was tested for association with chronotype using the CSM in a sample of Columbian adults and found that two subscales (“activity planning” and “morning alertness”) were associated but not total CSM score or the “morningness” subscale^22^. While the current work does not replicate or directly overlap those findings (i.e., a different, uncorrelated *PER2* SNP tested and different measure of chronotype), our findings and previous reports, taken together, suggest that circadian regulation of behavior and diurnal preference involves *PER2*, and as we now show, may involve circadian period.

## Conclusion

This is the first study to demonstrate a human genetic variant that associates with intrinsic circadian period. Our results show that the *PER2* rs35333999 T allele is associated with a longer intrinsic circadian period, which provides a likely underlying mechanism for the evening chronotype that was previously associated with this SNP. Remarkably, the single SNP explained 7% of the inter-individual differences in intrinsic circadian period (11-12 min). To put this into perspective, this difference in intrinsic circadian period conveyed by this single SNP is twice as large as the average difference in intrinsic circadian period found between men and women, which has been proposed to underlie sex differences in bedtimes, wake times, and sleep disturbances^41^. Differences in intrinsic circadian period and chronotype may also influence the susceptibility for diabetes and other metabolic disorders (https://www.ncbi.nlm.nih.gov/pubmed/30705391)^42,43^. Future studies will be necessary to reveal the molecular mechanism by which the genetic variant influences circadian period and chronotype and to determine its effect on other chronotype-associated physiologic or disease phenotypes.

## Materials and Methods

### UK Biobank sample

Study participants were from the UK Biobank prospective study (n = 503,325 adults aged 40–69 years), described in detail elsewhere^44^. For the current analysis, individuals of self-reported non-white ethnicity and relatives were excluded to avoid confounding effects. Phenotype and genotype data from 335,789 unrelated individuals of self-reported British white ancestry who gave informed consent for genetics were used in the current analysis. The UK Biobank study was approved by the National Health Service National Research Ethics Service (ref 11/NW/0382), all participants provided written informed consent to participate in the UK Biobank study, and procedures were conducted in accordance with the relevant guidelines and requirements.

### In-lab patient sample

The population sample consisted of 252 healthy men and women who had previously participated in inpatient physiological research protocols at the Brigham and Women’s Hospital between 2001 and 2011. Pre-study screening criteria for inclusion consisted of general good health, no medical or psychological/psychiatric conditions and no medications other than certain oral contraceptives; no shiftwork in the prior 3 years; and no travel across more than 1 time zone in the prior 3 months. Written informed consent was obtained from participants prior to enrollment in this genetic study and they were compensated for their participation. All study procedures received approval by the Partners Health Care Human Research Committee and were conducted in accordance to the *Declaration of Helsinki*.

### Phenotypes

#### Chronotype

In the UK Biobank sample, chronotype was self-reported by a touch-screen questionnaire at the baseline visit^12^. Chronotype was examined both as a categorical trait (n = 335,789) and a dichotomous trait, with definite morning responders set to control (n = 80,065) and definite evening responders set to case (n = 26,056).

In the in-laboratory sample, chronotype was assessed by MEQ^26^ from study participants prior to their admission to the laboratory. This 19-question survey was scored using published criteria and summed to obtain a composite score. MEQ composite score was used as a continuous variable in this analysis.

#### Sleep timing

For a subset of the inpatient laboratory studies, participants were required to maintain a stable, self-selected, 8-hour time-in-bed (TIB) schedule for 1–3 weeks prior to admission to the inpatient protocol. Adherence to this schedule was verified by several means including: wrist actigraphy, sleep diaries, and time-stamped daily call-ins at bedtimes and wake times. Bedtime, wake time, TIB duration, and midpoint of TIB, were determined from the average call-in times during the week (7 days) immediately prior to admission. Participants who did not maintain an 8-hour schedule were not included in the analysis of sleep timing.

#### In-laboratory circadian phenotypes

Some of the inpatient laboratory protocols assessed circadian phase and/or period using specific study procedures. Specifically, timing of the CBT_min_ and the dim light melatonin onset (DLMO) were used as circadian phase markers collected during constant routine or constant posture conditions (see^45^ and^46^ for detailed description). Intrinsic circadian period of CBT and of melatonin were assessed using forced desynchrony protocols^29,41,47,48^ as previously described^29,30^. Forced desynchrony protocols involved the collection of continuous CBT measurement and/or hourly blood sampling during a minimum of two weeks while participants maintained a sleep/wake cycle that was substantially greater than 24 hours (e.g., T = 28 or T = 42.85 hours). This allowed for the assessment of circadian measures under a T cycle to which the circadian system cannot entrain while light intensity was maintained at 0 lux during scheduled sleep and <15 lux during scheduled wakefulness. Additionally, circadian phase angle for CBT (difference between timing of CBT_min_ and wake time averaged from the week prior to admission) and melatonin (difference between DLMO and wake time) were determined.

### Genotypes

UKBiobank study: Genotyping was performed by the UK Biobank, and genotyping, quality control, and imputation procedures have been described^49^. In brief, blood was collected from participants, and DNA was extracted from the buffy coat samples. Participant DNA was genotyped on two arrays, UK BiLEVE and UKB Axiom with >95% common content and genotypes for ~800,000 SNPs were imputed to the Haplotype Reference Consortium reference panel^50^. We examined association of directly genotyped SNP rs35333999 and genotyped and well-imputed common variants (minor allele frequency >0.001 and imputation quality score >0.3) within a 400 kb region to capture multiple independent association signals.

In-laboratory Study: DNA was extracted from venous blood samples collected from each in-laboratory study participant. The *PER2* SNP rs35333999 was genotyped using the Sequenom platform that distinguishes allele-specific primer extension products by mass spectrometry (MALDI-TOF; Broad Institute, Cambridge, Massachusetts)^51^. Quality control and Hardy Weinberg Equilibrium were assessed using the genetic software PLINK^52^. An additional 58 African-American and Hispanic ancestry informative markers were genotyped using the same platform to identify the subset of subjects with genetic European ancestry and to correct for population stratification^53,54^.

### Association analysis

Association testing in both cohorts employed an additive genetic model of *PER2* rs35333999 T with self-reported chronotype or circadian measures using linear or logistic regression analysis in PLINK and adjustment for covariates: age, sex, and five principal components of ancestry. In the UK Biobank, single SNP association analysis to chronotype was performed for SNPs in a 400 kb region encompassing the *PER2* gene and included genotyping array as an additional covariate. Conditional genetic association analysis included further adjustment for SNPs (rs35333999 or regional lead SNP rs80271258) in regression models. P-values < 5 × 10^−8^ were considered significant. Analyses of in-laboratory participants of European ancestry (n = 196) as well as for the entire multiethnic sample (n = 252) were performed. In the multi-ethnic sample, an additional race/ethnicity covariate was included. P-values < 0.05 were considered significant for associations between *PER2* rs35333999 and circadian phenotypes. Sensitivity analysis was performed using the Welch’s t-test for unequal variances^55^ testing T allele carriers (TC and TT) vs CC homozygotes on covariate-adjusted residuals (age, sex, and 5 principal components of ancestry) for both melatonin and core-body temperature period. The additive effect of the TT genotype was not considered in this sensitivity analysis.

## Supplementary information

Supplementary Material

## Data Availability

The data that support the findings of this study from the UK BioBank will be made available at https://sleepgenetics.org and the underlying genotype and phenotype data are available through application to the UK Biobank. Other phenotype data are available on request, due to privacy or other restrictions, through co-corresponding author Dr. Scheer (fscheer@bwh.harvard.edu).
