# Supplementary material for: Chronotype Genetic Variant in PER2 is Associated with Intrinsic Circadian Period in Humans
Source: Sci Rep. 2019 Mar 29;9:5350. doi: 10.1038/s41598-019-41712-1 (PMC6440993; doi:10.1038/s41598-019-41712-1)

Chronotype Genetic Variant in *PER2* is Associated with

Intrinsic Circadian Period in Humans

Anne-Marie Chang, Jeanne F. Duffy, Orfeu M. Buxton, Jacqueline M. Lane, Daniel Aeschbach, Clare Anderson, Andrew C. Bjonnes, Sean W. Cain, Daniel A. Cohen, Timothy M. Frayling, Joshua J. Gooley, Samuel E. Jones, Elizabeth B. Klerman, Steven W. Lockley, Mirjam Munch, Shantha M.W. Rajaratnam, Melanie Rueger, Martin K. Rutter, Nayantara Santhi, Karine Scheuermaier, Eliza Van Reen, Michael N. Weedon, Charles A. Czeisler, Frank A.J.L. Scheer, and Richa Saxena

# Supplemental Figure Legends

**Supplemental Figure 1**. *PER2* regional association plot of results for continuous chronotype in unrelated participants of European ancestry from the UK Biobank. Results from a 400kb region around SNP rs35333999 are shown with chromosomal position on the x-axis. Filled circles show the -log10 P value (y-axis) from single SNP association analysis. The labeled SNP is shown in purple and additional SNPs in the locus are colored according to correlation (r^2^) with rs35333999 estimated by LocusZoom based on the CEU HapMap haplotypes. Genes within the region are shown in the lower panel. The blue line indicates the recombination rate.

**Supplemental Figure 2**. *PER2* regional association plot of results for continuous chronotype in unrelated participants of European ancestry from the UK Biobank. Results from a 400kb region around SNP rs35333999 are shown with chromosomal position on the x-axis. Filled circles show the -log10 P value (y-axis) from association analysis conditioned on regional lead SNP rs80271258. The labeled SNP is shown in purple and additional SNPs in the locus are colored according to correlation (r^2^) with rs35333999 estimated by LocusZoom based on the CEU HapMap haplotypes. Genes within the region are shown in the lower panel. The blue line indicates the recombination rate.

**Supplemental Figure 3**. Chronotype by *PER2* rs35333999 allele genotype in the multiethnic sample. Mean and individual MEQ scores for the T-allele carriers are shown by open symbols (square and circles, respectively). Mean and individual scores for the non-T allele group are denoted by filled symbols. The vertical line shows the standard error of the mean MEQ score for T-allele carriers.

**Supplemental Table 1.** Phenotype measures by *PER2* rs35333999 genotype and results from association testing with circadian phenotypes in the entire multiethnic sample. Phenotype means (SD) and the number of males/females are listed by rs35333999 genotype: T-allele carriers and non-T carriers. Significant results (p<0.05) are shown in bold.

| **Phenotype** | **n** | **Mean (SD)** | **n** | **Mean T**  **Carriers** | **n** | **Mean Non-T Carriers** | **Beta (SE)** | | **p** | |  |
| --- | --- | --- | --- | --- | --- | --- | --- | --- | --- | --- | --- |
| Age (years) | 252 | 27.33 (11.45) | 14 | 26.07 (4.92) | 238 | 27.41 (11.73) | | ----- | | ----- | |
| Sex (M/F) | 252 | 164/88 | 14 | 9/5 | 238 | 155/83 | | ----- | | ----- | |
| MEQ score | 246 | 52.70 (11.68) | 13 | 46.38 (14.89) | 233 | 53.05 (11.42) | | -4.43 (2.84) | | 0.120 | |
| CBT period (h) | 77 | 24.17 (0.20) | 7 | 24.35 (0.16) | 70 | 24.15 (0.19) | | 0.19 (0.08) | | **0.022** | |
| Mel period (h) | 68 | 24.18 (0.19) | 6 | 24.33 (0.16) | 62 | 24.16 (0.18) | | 0.16 (0.08) | | 0.051 | |
| CBT phase (clock h) | 106 | 5:04 (2:04) | 6 | 4:53 (1:58) | 100 | 5:05 (2:05) | | 0.09 (0.87) | | 0.917 | |
| Mel phase (clock h) | 120 | 22:28 (1:52) | 5 | 22:49 (1:32) | 115 | 22:27 (1:53) | | 0.57 (0.83) | | 0.493 | |
| CBT phase angle (h) | 104 | -3.08 (1.33) | 6 | -2.88 (1.08) | 98 | -3.09 (5.44) | | 0.36 (0.57) | | 0.527 | |
| Mel phase angle (h) | 118 | -9.45 (1.18) | 5 | -9.06 (1.10) | 113 | -9.46 (1.18) | | 0.55 (0.53) | | 0.302 | |
| Bedtime (clock h) | 189 | 23:58 (1:26) | 10 | 0:34 (1:52) | 179 | 23:56 (1:24) | | 0.74 (0.43) | | 0.090 | |
| Wake time (clock h) | 189 | 8:01 (1:26) | 10 | 8:35 (1:51) | 179 | 7:59 (1:25) | | 0.69 (0.44) | | 0.115 | |
| TIB duration (h) | 189 | 8.05 (0.14) | 10 | 8.01 (0.16) | 179 | 8.05 (0.14) | | -0.05 (0.05) | | 0.314 | |
| TIB midpoint (clock h) | 189 | 3:59 (1:26) | 10 | 4:35 (1:51) | 179 | 3:57 (1:25) | | 0.72 (0.44) | | 0.101 | |

**Supplemental Figure 1.**

rs35333999 *PER2* region signal


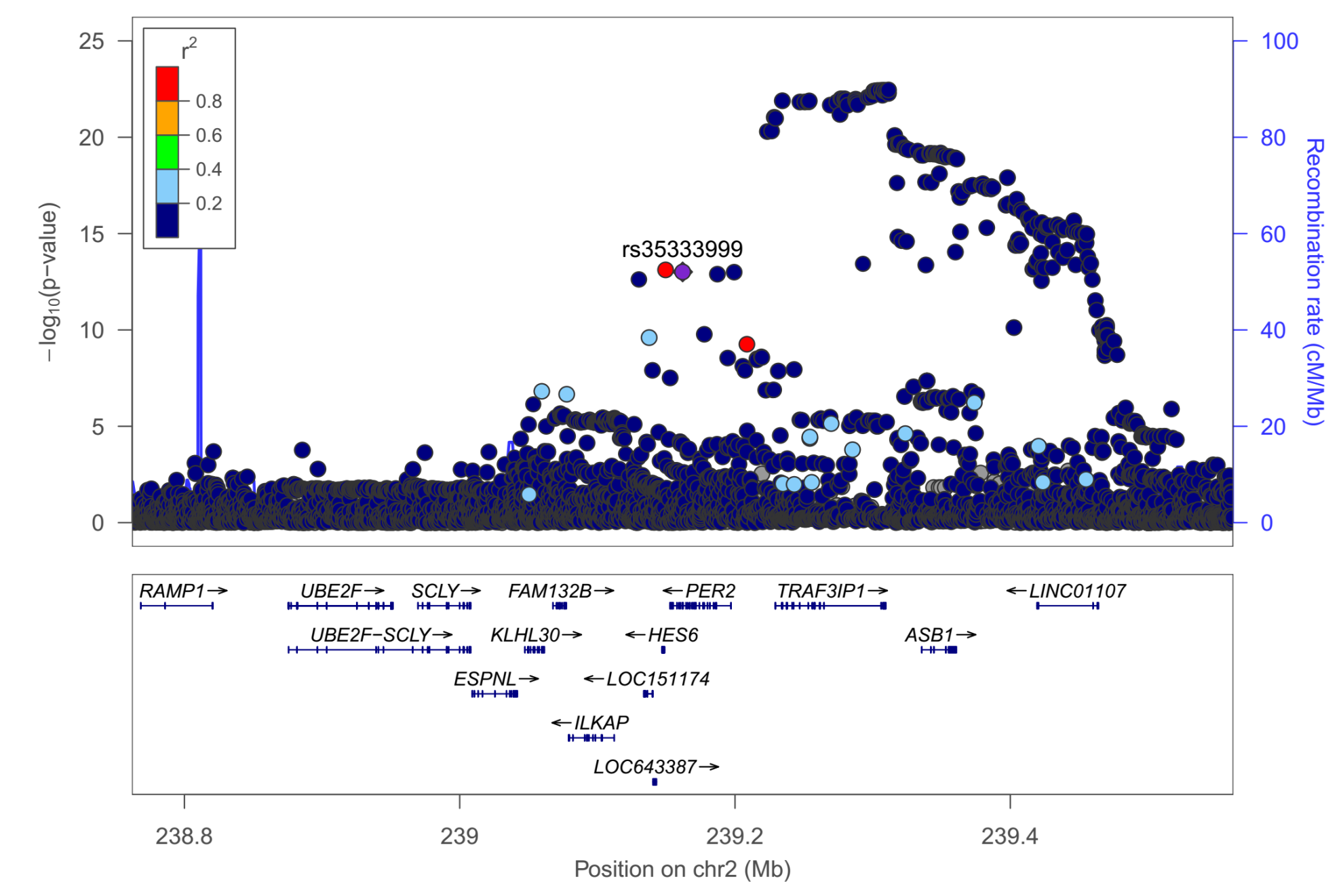


**Supplemental Figure 2.**

rs35333999 signal adj for rs80271258


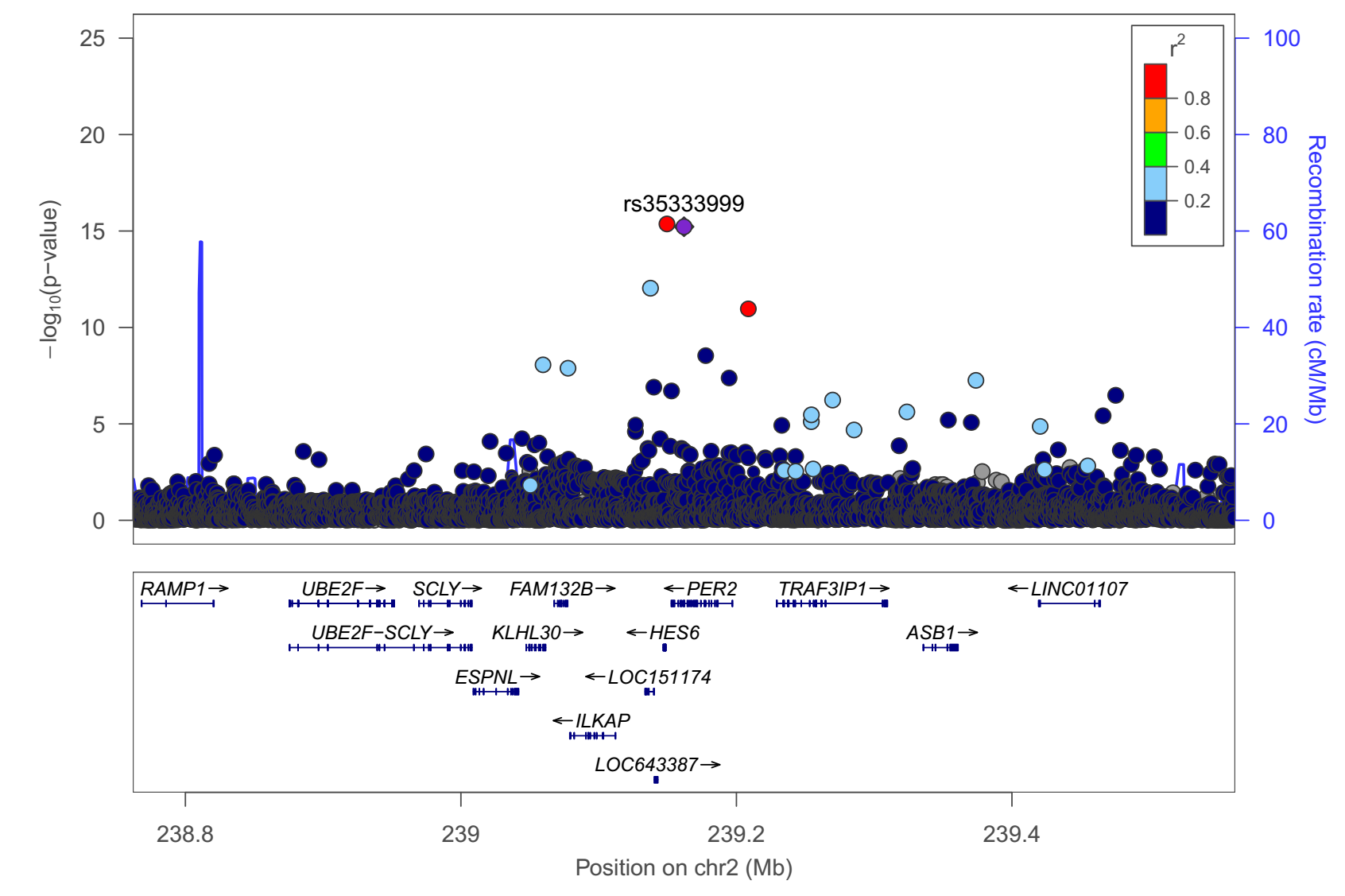


**Supplemental Figure 3.**


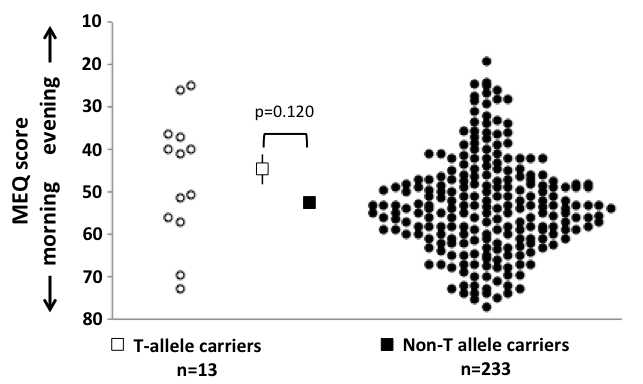

Supplement: Supplementary file 1 — Supplementary Material [file 41598_2019_41712_MOESM1_ESM.docx]
